# Rheumatoid arthritis mediator CD18 expression by *Staphylococcus aureus* superantigen C in rats

**Published:** 2019-08

**Authors:** Mahsa Banaei, Gholam Hosein Alishiri, Ramezan Ali Ataee, Ardeshir Hesampoor Mahalati

**Affiliations:** 1 Applied Microbiology Research Center, Baqiyatallah University of Medical Sciences, Tehran, Iran; 2 Department of Biology, Central Tehran Branch, Islamic Azad University, Tehran, Iran; 3 Department of Medical Microbiology, School of Medicine, Baqiyatallah University of Medical Sciences, Tehran, Iran; 4 Department of Rheumatology, School of Medicine, Baqiyatallah University of Medical Sciences, Tehran, Iran; 5 Chemical Injuries Research Center, Baqiyatallah University of Medical Sciences, Tehran, Iran

**Keywords:** Staphylococcal superantigen, Rheumatoid arthritis, Cd18 lymphocytes, Biomarker

## Abstract

**Background and Objectives:**

Microbial superantigens have been reported in the blood and synovial fluid of rheumatoid arthritis patients, raising the question of whether the presence of these superantigens could provoke the induction of inflammatory biomarkers expression or not. The purpose of this study was to examine the *Staphylococcus aureus* superantigen C on CD18 expression.

**Materials and Methods:**

The superantigen C was purified by ultrafiltration. Immunoblotting was performed using a specific antibody. Also, 50 micrograms of superantigens (toxin) were injected intraperitoneally and intra-articularly into separate rat groups. Blood was collected and RNA extracted. Then, the cDNA was synthesized. The expression of CD18 marker was evaluated using RT-real-time PCR, and the results were descriptively analyzed.

**Results:**

The results of this study revealed that 50 μg of toxin, injected intra-articularly and intraperitoneally, showed the surplus expression of the marker CD18 in the blood of rats after 20 days. By this method, the expression of the marker CD18 was significantly different between rats that received the superantigen intra-articularly and intraperitoneally (2.10; 2.3 and 3.3 folds) and the controls (P≤ 0.05).

**Conclusion:**

The results indicated that the presence of *Staphylococcal* of superantigen C in the body of rats has enhanced the expression of the CD18 inflammatory marker more than 3 times. This valuable finding is an introduction to further research and could provide new methods to prevent and control inflammatory diseases, including rheumatoid arthritis.

## INTRODUCTION

CD molecules (cluster differentiation or classification determinant) are cell surface molecules which are expressed on various cell types in the immune system and their role is to regulate the cellular activity of the organisms (1). Various roles have been mentioned for CD18 ligand; however, further studies should be conducted to discover other roles of CD18 ligand (2). The most widely studied subfamily of beta 2 integrin is CD11a/CD18, CD11b/CD18, and CD11c/CD18 (3). The role of these adhesion molecule expressions during the progression of rheumatoid arthritis (RA) disease by adjuvant-induced arthritis (AIA) was reported in rats (4). Research has shown that the subfamily of beta integrin (CD18) is implicated in macrophage fusion and acts as a hallmark of chronic inflammatory disorder (5). Moreover, recent research has revealed that the neutrophil migration from blood into inflamed tissues is mediated by adhesion molecules on neutrophils and on vascular endothelium (6). The factors that increase the expression of CD18 glycoproteins may be contributed to inflammation and tissue destruction. While the increase in cellular marker of CD18 and inflammatory diseases, including rheumatoid arthritis, is unclear in humans, particularly in patients with RA, this marker is associated with an increase; however, the endogenous or exogenous origin to express CD18 is not fully understood. Nevertheless, the changes of soluble CD18 in murine autoimmune arthritis and RA have been shown as consequences of treatment response (7). Furthermore, one of the important roles of CD18 is that the existence of this biomarker is essential for CD4, CD25, and T regulatory cells activation (8). In addition, phenotypically, changes in monocytes/macrophages, which expressed the CD18 mRNA in peripheral blood and synovial fluid of patients with RA, have been illustrated (9). Various biological and environmental agents, such as classical superantigens (10–12), were traced in the blood and synovial fluids of patients with RA. These agents may induce the expression of various cytokines, and thus in recent years, effective biological therapies have been developed. However, the specific role of antigens in stimulating the expression of CDs markers in developing inflammatory diseases is not still clear. The aim of this study was to examine the effects of staphylococcal superantigen C on CD18 marker expression in laboratory rats using RT-real-time PCR.

## MATERIALS AND METHODS

Bacterial culture media and electrophoresis materials were purchased from Merck, Germany, Millipore filters (0.45 micrometers) from Sartorius Company, and the Amicon ultra centrifugal filter device 10, 30, 50 and 100 kDa systems from Millipore Corporation. Also, mono specific polyclonal antibody type C (RbPAb Enterotox in C 500 μg in 1mg / mL, Ab *Staphylococcus aureus* C 15897, Abcam) was purchased. A Wistar laboratory animal rat, with a weight of 250 to 300 grams, was purchased from the animal center of Baqiyatallah University of Medical Sciences. RNA extraction kit and cDNA synthesis kit were purchased by the representative of MN and Takara companies.

### Preparation of *Staphylococcus aureus* superantigen C: Bacterial strain and culture conditions.

In this study, an enterotoxin C producer of standardized strain of *S. aureus* was used (13). The bacterium was inoculated into sterilized BHI-broth medium and incubated for 24 hours at 37°C; then, as preculture, it was inoculated into 1 liter of culture medium (0.5% v/v) and incubated at 37°C for 24 hours. Then, the above culture was transferred into the decanter and placed in a refrigerator at 5°C for 48 hours and then for 5 days at laboratory temperature. Then, the precipitate was collected. To isolate the bacterial cells, centrifuge was performed (5,000 ×g at 5°C for 5 minutes); and the supernatant was sterilized by filtration (0.45 μm). The sequential ultra-filtration was centrifuged (6000 ×g at 5°C for 10 to 15 minutes). Also, the output of the filter 100 KDa was centrifuged with a 50 KDa filter and its output with a 30 KDa filter; then, the output was filtered with 10 kDa. This procedure was repeated 10 times; finally, 5 mL of an extract of 10 kDa filter was collected and stored at refrigerated temperature for further study.

### Determination of protein concentration.

To determine the concentration of extracted protein at different stages of production and purification, a Nano Drop was used at 280 nm wavelength with standard protein solution. In all cases, the sterilized extract of BHI-broth medium was used as a blank.

### SDS-electrophoresis and immunoblot.

To determine the isolation of toxin from the extracted solution, SDS-electrophoresis was performed with 15% polyacrylamide gel; then, immunoblotting was performed.

### Superantigen injection into the animal and sampling.

In this study, 4 groups of 5 Wistar rats, weighing 250 to 300 grams, were selected.

Group I: 50μL solution containing 1 mg of superantigen C was injected into the first group of rats intra-articularly with insulin syringe.
Group II: 50 μL of solution containing 1 mg of superantigen was injected into the second group intraperitoneally with insulin syringe.
Group III: 50 μL of normal saline solution was injected into the third group by intraperitoneal injection.
Group IV: The members of the fourth group received no injection (control group).

### Blood sampling.

Because blood sampling through the tail vein of the rats was difficult, it was not possible to take blood samples from the tail; therefore, the animals were anaesthetized by ketamine hydrochloride (1 to 1.5 mL of 50 mg/10 mL) and under aseptic conditions, blood was taken from the heart of the animals. Thus, at time zero, only blood sampling was done from the control group. After injections, blood sampling was done on days 10, 20, 40 and 50 from each group at each stage and RNA was immediately extracted.

### Extraction of RNA from rat blood.

To extract RNA, a kit (NucleoSpin RNA blood, Takara Japan, Ref; 740200.50) was used. In this kit, it was instructed to use liquid Proteinase K. The RNA was extracted and RT-real-time PCR was performed.

RNA extraction steps were performed in an environment free of any contamination and at temperature of 25°C. In brief, the extraction steps were as follow.

### Measuring the concentration of extracted RNA.

RNA was extracted to determine its amount of lipid, protein, any contamination and DNA using spectrophotometric method of NanoDrop device. Graphs and OD content (Optical Density) of each sample was recorded by the device.

### Primer designing.

The relevant CD sequence gens (rat type), such as CD4+, CD8+ and CD18+, was selected from the database of the gene bank. The pair primers were designed based on the genes using available software (Online Gene script and Primer3 software).

### cDNA Synthesis.

For total cDNA synthesis, all mRNAs of the samples were used as template for synthesis by Takara cDNA synthesis kit and proprietary primer.

According to the kit direction for cDNA synthesis, first, the initial calculations for the normalization of the selected samples and the uniform concentration of the total RNA extracted from the samples were performed.

### RT-Real time PCR method.

To investigate the rate and level of expression of inflammatory markers (CD4, CD8 and CD18), each of the samples and the housekeeping reference gene were used by RT-real-time PCR.

RT-real-time PCR was used for quantitative analysis of gene expression using Takara kit with SYBR Premix (Ex TaqTM II (Tli Plus) and Cat. No-Brand: RR820Q-Takara).

For each of the CD4, CD8 and CD18 markers, forward and reverse primers were synthesized. They contained 6 vials and kept at the laboratory temperature outside the refrigerator. According to the instructions provided by the manufacturer, to use any primer with a concentration of 10–12 mole, the amount of RNase free distilled water for each vial had to be 300 μL.

To use primers, a concentration of 100 μmoles was reduced with a ratio of 1 to 10 (for each vial, 9 water and 1 primer), using dilution, to a concentration of 10 or 12 μmole. Then, according to the instructions of the Takara kit and for ease of use, 0.8 μL of the concentration of 10–12 μmole of the forward primer and 0.8 μL of the 10–12 μmole concentration of the reverse primer were mixed for each marker. Also, the GAPDH reference gene primers with a concentration of 10 to 12 μmole per 50 μL was also diluted and the primers F and R were mixed together in a defined proportion.

### RT- real-time PCR mixture preparation method and protocol.

All preparation steps for RT-real-time PCR were done in aseptic conditions and sterile instruments.

Four rows of 12 cap strips were selected and marked: The first 12 horizontal stripes of cap strips for CD4 examination, the second row for CD8, the third for CD18, and the fourth for evaluating the gene encoded the glyceraldehyde 3-phosphate dehydrogenase (GAPHD) as reference gene.

1- Transferring 10 μL SYBR Premix Ex TaqTM II (2×) into each of the microtubules attached to the Cap Strip
2- Transferring 1 μL of a cDNA solution synthesized as a template (<100 ng)
3- Transferring 1.6 μL of mixed primers PCR forward primer (10μM) and PCR reverse primer (10μM) for each of the desired markers in each row.
4- Adding 0.4 μL of ROX Reference Dye (50×) or Dye II (50×)

Each vial was mixed gently, and by adding sterilized distilled water (dH2O), each of the reaction tubes reached 20 μL. Then, after a short spin of cap strips containing the sample and placing them in the real-time device vertically, the apparatus was prepared at 95°C for 3 minutes, with a denaturation of 1 minute at 95°C, annealing 60% for 1 minute, and an extension.

After the reaction, CT data for each sample in each vial were determined using the appropriate formulas for the level of expression of each marker. First, the average of all CTs and the mean of ΔCTs were calculated; then, by subtracting the ΔCT of the control and ΔCT of each of the samples, the expression level of each of the genes was determined and categorized using the formula of 2-ΔCT=ΔΔCT= gene expression level (14).

## RESULTS

The activation of *Staphylococcus aureus*, the producer of superantigen C (Type C enterotoxin), was associated with the growth and opacity of the culture. The turbidity obtained from the media with optical density spectrophotometer and at a wavelength of 560 nm was found to be 0.53.

Also, the inoculation of bacterial preculture into BHI-broth supplemented media was associated with mass production and bacterial growth.

The results of sequential ultrafiltration showed that the highest amount of protein with superantigen C property of the extracted protein was obtained under ultrafilter 30 kDa.

The concentration of extracted protein, was approximately 21 mg from 1 liter of bacterial culture.

The results of SDS-PAGE electrophoresis showed the optimal procedure for the isolation of *S. aureus* superantigen C, which was confirmed by immunoblotting.

The results of the preparation concentration of the superantigen C of *S. aureus* was obtained from 1 liter of culture medium containing about 3 mL of toxin solution at a concentration of 7 mg/mL, which was diluted as 1 mg / mL. The results of macroscopic assay revealed that the effect of superantigens on the liver, spleen, and kidneys are demonstrated in Table 1.

**Table 1.** Sampling time and macroscopic signs and symptoms of the rats’ organs

**Clinical signs and symptoms of rats**	**Spleen**	**Kidney**	**Liver**	**Knee joint**	**Blood**	**Injection type**
Healthy	Normal	Normal	Normal	Normal	Transparent	Controlgroups
Diarrhea, total liver, spleen, and kidney weight was 27 g of mice with, the mean of weight being 351 g	Getting smaller	Normal	Swollen	Swollen	Transparent	Groups received intra-articular injection
Diarrhea, relatively untidiness, was difficult to bleed and was quickly clotted, total liver and spleen and kidneywere 24 g of mice’ weight, with average weight of 317 g	Getting smaller	Swollen	Swollen	Swollen	Dark	Groups received intraperitoneal injection
Normal and Healthy	Normal	Normal	Normal	Normal	Transparent	Groups received intraperitoneal saline injections

### RNA extraction result.

The results of extracted RNA, using NanoDrop, in a wavelength of 260 *nm* for each sample and electrophoresis in 1% gel, showed the appropriate procedure.

### cDNA Synthesis.

The results of the cDNA synthesis in all extracted RNA samples are used for RT-real-time PCR reaction amplification.

### Primer design.

The results of primers design were as CD18+ Rat Forward: 5′ CAGGAATGCACCAAGTACAAAGT 3′ and CD18+ Rat Reverse: 5′ CCTGGTCCAGTGAAGTTCAGC 3′, which was commissioned with the advice of a bioinformatics expert.

**Fig. 1:**
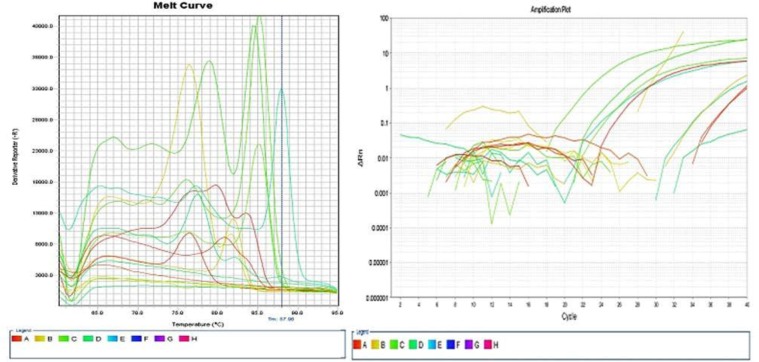
The results of amplification of 12 samples for CD18 and housekeeping genes The blue color is associated with the CD4 marker, the yellow color with the CD8 marker, the violet green color with the marker CD18, and the red color with the housekeeping gene (Gap HD).

The results of RT-real-time PCR reaction amplification of the 12 samples are shown in Fig. 1 as amplification plot and melted curve. The results of the RT-real-time PCR reaction are represented graphically (Fig. 1).

Based on Ct data, each CD sample and housekeeping gene expression are arranged as follow:

The mean of Ct negative control, intra-articular and intraperitoneal received superantigens and the ΔCt and also the expression rate of each of the inflammatory markers is presented in Table 2. The results of data analysis are presented in Table 3.

**Table 2.** Shows the rate of expression of CD markers and the housekeeping genes.

**Gene**	**Mean Ct of C Sample**	**Mean Ct of IA Sample**	**Mean Ct of IP Sample**	ΔCt **of IA Sample**	ΔCt **of IP Sample**	ΔΔCt **of IA Sample**	ΔΔCt **of IP Sample**
CD4	34.41	33.31	34.49	1.1	0.08	2.14	0.94
CD8	33.93	32.79	32.31	1.14	1.62	2.20	3.07
CD18	25.42	25.70	25.71	0.28	0.29	0.82	0.81
GAPHD	35.54	26.78	33.17	2.76	2.37	4.433	5.16

Control = C; intra-articular = IA; intraperitoneal = IP; Cycle threshold=Ct

**Table 3.** Analysis of the data obtained from RT- real-time PCR by Kruskal-Wallis test and Mann-Whitney test

	**Groups**			**p-value**	**Multiple comparison**

	**1. Negative Control**	**2. Intra articular SAg Received**	**3. Intra peritoneal SAg Received**		
CD4	33.29±1.65	35.69±1.88	35.54±1.03	0.19	(1vs.2, p=0.27);(1vs.3, p=0.05);(2vs.3, p=0.83)
CD8	33.47±.73	31.52±4.76	35.09±1.77	0.29	(1vs.2, p=0.51);(1vs.3, p=0.13);(2vs.3, p=0.27)
CD18	25.25±.75	22.69±1.65	27.95±3.35	0.04	(1vs.2, p=0.05);(1vs.3, p=0.13);(2vs.3, p=0.05)
House kipping Gene	33.06±1.86	32.36±.69	32.52±.79	0.83	(1vs.2, p=0.77);(1vs.3, p=0.99);(2vs.3, p=0.44)

As presented in Table 2, the injection of superantigen C of *S. aurous* has been shown to increase the expression of all the above genes. The results of data analysis presented in Table 3 indicate that intra-articular injection of this superantigen stimulate the expression of CD4 over intra-peritoneal injection, while injection in both cases was associated with an increase in CD18 expression.

## DISCUSSION

Evidence suggests that CD18 plays a vital role in cellular activities. In some reports, the name of CD18 has changed to Beta- 2 integrin, and investigation in genetic bases revealed that the beta- 2 integrin (CD18) is organized into 16 exons of about 40 kb (15). Furthermore, some reports suggest that the complex receptors CD11a/CD18 is a subunit of leukocyte function associated Ag-1 (LFA-1), which has shown a crucial role in T-cell/endothelial cell interactions (16). Another study found that lack of CD18 on either cell type leads to dramatically reduced TGF-β1 release by macrophages, which is due to defective adhesion and subsequent impaired phagocytic clearance of neutrophils (17) and possibly vice versa. This fact demonstrates that the secretion of TGF-bata-1 is essential for wound-healing and anti-inflammatory properties. Although the precise role of CD18 in the pathogenesis of inflammatory diseases, such as RA, is still unclear, gene structural studies showed different complexities on CD11/CD18 (18); However, the CD18 mutant mice model has been analyzed for the role of leukocyte-integrin-dependent adhesion in inflammatory diseases (19). In this method, using anti-CD18 antibodies to influence neutrophil activation was reported (20). In fact, the high levels of CD18 expression as a key role of RA pathogenesis by involving leukotriene B4 were shown. Therefore, inhibiting ex-vivo leukotriene B4 (LTB 4), which induced Mac-1 (CD11b/CD18) expression in leucocytes of patients with RA disease, could change the clinical signs and symptoms of the RA disease (21). However, factors affecting the expression of high level of CD18 have not been directly reported.

Although the term autoimmune has been challenged with RA, there are numerous reports of presence of different superantigens in blood (10), synovial fluid or both (11) in patients with RA. Recently, a research has assayed wide members of a superantigen family (PTSAg) in urine samples in patients with RA (22) and reported the presence of several bacterial superantigens in the urine of these patients. Another researcher reported inflammation with initial dominance of CD8 T-cells in rats after intra-articular *Staphylococcal* superantigen A injection (23).

Accordingly, in the present study, it was hypothesized that the first stage involved in inflammation and the superantigen C *S. aureus* may provoke the induction of CD18 expression.

Thus, the effect of purified superantigen C on the induction of expression biomarker CD18 was examined in rats. The results revealed that 50 μg of toxin injected intera-articularly and intraperitoneally showed the surplus expression of the marker of CD18 in blood of the rats after 20 days. Using this method, the expression of the markers in rats that received superantigen intra-articularly and intraperitoneally was 3 times higher than the controls. This finding indicated that the presence of superantigen C of *S. aureus* induced expression of the CD18 inflammatory markers. This valuable finding is an introduction to further research and may help to prevent and control inflammatory diseases, including RA.

In the present study, although only 1 superantigen was applied, it is clear that other bacterial superantigens may have similar effects, as in our previous research, 5 classical *Staphylococcal* superantigens were found in synovial fluids of patients with rheumatoid arthritis (30). In addition, in recent years, the role of microbiome in rheumatoid arthritis pathogenesis was considered (24–26). However, microorganisms naturally inhabit various sites of the human body, and thus new molecular diagnostic methods may help to clarify the role of bacterial superantigens in inflammatory diseases, especially RA.

The clinical implication of current study is that a possible role of superantigen in clarification the mechanisms of pathophysiology of RA disease. The CD18 biomarkers expression in this study was influenced by superantigen C, and it has been the basic tool for drug evaluation and patient management. Furthermore, controlling CD18 biomarker expression by exogenous superantigens may help develop protocols for designing diagnostic methods and prevention procedures.

## References

[1] Hardcastle SL Brenu EW Johnston S Nguyen T Huth T Wong N Characterisation of cell functions and receptors in chronic fatigue syndrome/myalgic encephalomyelitis (CFS/ME). BMC Immunol 2015;16:35. 26032326 10.1186/s12865-015-0101-4 PMC4450981

[2] Boyd AJ Rubin BB Walker PM Romaschin A Issekutz TB Lindsay TF A CD18 monoclonal antibody reduces multiple organ injury in a model of ruptured abdominal aortic aneurysm. Am J Physiol 1999;277(1):H172–182. 10409195 10.1152/ajpheart.1999.277.1.H172

[3] Elangbam CS Qualls CW Dahlgren RR Jr. Cell adhesion molecules--update. Vet Pathol 1997;34:61–73. 9150551 10.1177/030098589703400113

[4] Halloran MM Szekanecz Z Barquin N Haines GK Koch AE Cellular adhesion molecules in rat adjuvant arthritis. Arthritis Rheum 1996;39:810–819. 8639178 10.1002/art.1780390514

[5] Podolnikova NP Kushchayeva YS Wu Y Faust J Ugarova TP The role of integrins alpha M beta2 (Mac-1, CD11b/CD18) and alphaDbeta2 (CD11d/CD18) in macrophage fusion. Am J Pathol 2016;186:2105–2116. 27315778 10.1016/j.ajpath.2016.04.001 PMC4973655

[6] Birner U Issekutz TB Issekutz AC The role of selectins in VLA-4 and CD18-independent neutrophil migration to joints of rats with adjuvant arthritis. Eur J Immunol 1999;29:1094–1100. 10229075 10.1002/(SICI)1521-4141(199904)29:04<1094::AID-IMMU1094>3.0.CO;2-6

[7] Kragstrup TW Jalilian B Keller KK Zhang X Laustsen JK Stengaard-Pedersen K Changes in soluble CD18 in murine autoimmune arthritis and rheumatoid arthritis reflect disease establishment and treatment response. PLoS One 2016;11(2):e0148486. 26849368 10.1371/journal.pone.0148486 PMC4743942

[8] Marski M Kandula S Turner JR Abraham C CD18 is required for optimal development and function of CD4+CD25+ T regulatory cells. J Immunol 2005;175:7889–7897. 16339524 10.4049/jimmunol.175.12.7889

[9] Highton J Carlisle B Palmer DG Changes in the phenotype of monocytes/macrophages and expression of cytokine mRNA in peripheral blood and synovial fluid of patients with rheumatoid arthritis. Clin Exp Immunol 1995;102:541–546. 8536370 10.1111/j.1365-2249.1995.tb03850.x PMC1553364

[10] Ataee RA Golmohammadi R Alishiri GH Mirnejad R Najafi A Esmaeili D Simultaneous detection of *Mycoplasma pneumoniae*, *Mycoplasma hominis* and *Mycoplasma arthritidis* in synovial fluid of patients with rheumatoid arthritis by multiplex PCR. Arch Iran Med 2015;18:345–350. 26058928

[11] Rahdar HA Golmohammadi R Mirnejad R Ataee RA Alishiri GH Kazemian H Diversity of virulence genes in *Brucella melitensis* and *Brucella abortus* detected from patients with rheumatoid arthritis. Microb Pathog 2018;118:247–250. 29578063 10.1016/j.micpath.2018.03.034

[12] Zahiri Yeganeh S Ataee RA Alishiri GH Movahedi M Bacteriological and molecular assessment of *Staphylococcal* enterotoxin e in the blood of patients with rheumatoid arthritis. Jundishapur J Microbiol 2015;8(2): e16621. 25793096 10.5812/jjm.16621 PMC4353035

[13] Ataee RA Kamali M Karami A Ghorbani M Standardization of molecular method detection of the ent C in *S. aureus* isolated from human infections and determine it’s sequence. J Mil Med 2012; 14: 226–234.

[14] Yuan JS Reed A Chen F Stewart CN Statistical analysis of real-time PCR data. BMC Bioinformatics 2006;7:85 16504059 10.1186/1471-2105-7-85 PMC1395339

[15] Weitzman JB Wells CE Wright AH Clark PA Law SK The gene organisation of the human beta 2 integrin subunit (CD18). FEBS Lett 1991;294:97–103. 1683838 10.1016/0014-5793(91)81351-8

[16] Kavanaugh AF Lightfoot E Lipsky PE Oppenheimer-Marks N Role of CD11/CD18 in adhesion and transendothelial migration of T cells. Analysis utilizing CD18-deficient T cell clones. J Immunol 1991;146:4149–4156. 1710241

[17] Peters T Sindrilaru A Hinz B Hinrichs R Menke A Al-Azzeh EA Wound-healing defect of CD18(−/−) mice due to a decrease in TGF-beta1 and myofibroblast differentiation. EMBO J 2005;24:3400–3410. 16148944 10.1038/sj.emboj.7600809 PMC1276170

[18] Gjelstrup LC Boesen T Kragstrup TW Jorgensen A Klein NJ Thiel S Shedding of large functionally active CD11/CD18 Integrin complexes from leukocyte membranes during synovial inflammation distinguishes three types of arthritis through differential epitope exposure. J Immunol 2010;185:4154–4168. 20826754 10.4049/jimmunol.1000952

[19] Wilson RW Ballantyne CM Smith CW Montgomery C Bradley A O’Brien WE Gene targeting yields a CD18-mutant mouse for study of inflammation. J Immunol 1993;151:1571–1578. 8101543

[20] Saltzman WM Livingston TL Parkhurst MR Anti-bodies to CD18 influence neutrophil migration through extracellular matrix. J Leukoc Biol 1999;65:356–363. 10080540 10.1002/jlb.65.3.356

[21] Alten R Gromnica-Ihle E Pohl C Emmerich J Steffgen J Roscher R Inhibition of leukotriene B4-induced CD11B/CD18 (Mac-1) expression by BIIL 284, a new long acting LTB4 receptor antagonist, in patients with rheumatoid arthritis. Ann Rheum Dis 2004;63:170–176. 14722206 10.1136/ard.2002.004499 PMC1754875

[22] Grace LE Bukhari M Lauder RM Bishop LA Taylor AM The Presence of staphylococcal toxins in the urine of patients with rheumatoid arthritis. Ann Rheum 2016; 75(supple 2): 930.

[23] Gerlach K Tomuschat C Finke R Staege MS Brutting C Brandt J Experimental arthritis in the rat induced by the superantigen *Staphylococcal* enterotoxin A. Scand J Immunol 2017;85:191–196. 28128856 10.1111/sji.12530

[24] Roszyk E Puszczewicz M Role of human microbiome and selected bacterial infections in the pathogenesis of rheumatoid arthritis. Reumatologia 2017;55:242–250. 29332963 10.5114/reum.2017.71641 PMC5746635

[25] Scher JU Abramson SB The microbiome and rheumatoid arthritis. Nat Rev Rheumatol 2011;7:569–578. 21862983 10.1038/nrrheum.2011.121 PMC3275101

[26] Nogueira AR Shoenfeld Y Microbiome and autoimmune diseases: cause and effect relationship. Curr Opin Rheumatol 2019;31:471–474. 31192811 10.1097/BOR.0000000000000628

